# Neurodevelopmental disease-causing variants in choline kinase *CHKA* gene couple phosphatidylcholine synthesis to oxidative stress damage and disease etiology

**DOI:** 10.1016/j.jbc.2025.110983

**Published:** 2025-11-25

**Authors:** Mahtab Tavasoli, Mariam Alkandari, Gabriel Dorighello, Michael McPhee, Neale D. Ridgway, Kathy Isaac, Stanislav Sokolenko, Reza Maroofian, Anju Shukla, Maha S. Zaki, Henry Houlden, Christopher R. McMaster

**Affiliations:** 1 Department of Pharmacology, Dalhousie University, Halifax, Nova Scotia, Canada; 2 AGADA Biosciences, Inc., Halifax, Nova Scotia, Canada; 3 Department of Biochemistry and Molecular Biology, Dalhousie University, Halifax, Nova Scotia, Canada; 4 Department of Process Engineering & Applied Science, Dalhousie University, Halifax, Nova Scotia, Canada; 5 Department of Neuromuscular Diseases, UCL Queen Square Institute of Neurology, London, UK; 6 Department of Medical Genetics, Kasturba Medical College, Manipal, Manipal Academy of Higher Education, Manipal, India; 7 Department of Clinical Genetics, Human Genetics and Genome Research Institute, National Research Centre, Cairo, Egypt; 8 Department of Neuromuscular Diseases, Institute of Neurology, University College London, London, UK

**Keywords:** phospholipid, phosphatidylcholine, metabolism, inherited disease, mitochondria

## Abstract

Biallelic variants in *CHKA*, which encodes the first enzyme in the CDP-choline pathway for the synthesis of phosphatidylcholine, cause an inherited disorder characterized by epilepsy, microcephaly, and intellectual disability. How a deficiency in CHKA activity manifests these neurological symptoms is poorly understood. In this study, we investigated patient-derived fibroblasts with *CHKA* missense variants to elucidate the molecular and biochemical mechanisms underlying the associated pathologies. *CHKA* variant fibroblasts exhibited impaired phospholipid and triacylglycerol synthesis, altered mitochondrial morphology and function, elevated reactive oxygen species (ROS) levels, and increased lipid peroxidation, suggesting a mechanism by which defective CHKA activity leads to lipid damage. Treatment with FCCP, a mitochondrial uncoupler, reduced ROS levels and attenuated lipid peroxidation in *CHKA* patient fibroblasts, suggesting a potential approach to therapeutic intervention.

The Kennedy pathway is the primary route for the synthesis of phosphatidylcholine (PC), the major phospholipid in most mammalian cellular membranes (1, 2, 3, 4, 5). PC is essential for membrane integrity and structure, the function of membrane-embedded proteins, and serves as a source of numerous second messengers (2, 5, 6). The initial step in the CDP-choline pathway is the phosphorylation of choline to produce phosphocholine (7, 8, 9, 10, 11, 12) catalyzed by choline kinase α and β and encoded by the *CHKA* and *CHKB* genes, respectively (4, 7, 8, 9, 10, 11, 13, 14, 15, 16, 17, 18, 19, 20, 21, 22, 23). In the next step of the pathway, phosphocholine is converted to CDP-choline by CTP: phosphocholine cytidylyltransferase α and β encoded by the *PCYT1* and *PCYT2* genes (24, 25, 26, 27, 28, 29, 30, 31, 32, 33, 34, 35, 36, 37, 38). Lastly, two cholinephosphotransferases encoded by *CHPT1* and *CEPT1* catalyze the condensation of CDP-choline with diacylglycerol to produce PC (13, 39, 40, 41, 42, 43, 44, 45, 46, 47, 48, 49) (Fig. 1*A*).

**Figure 1:**
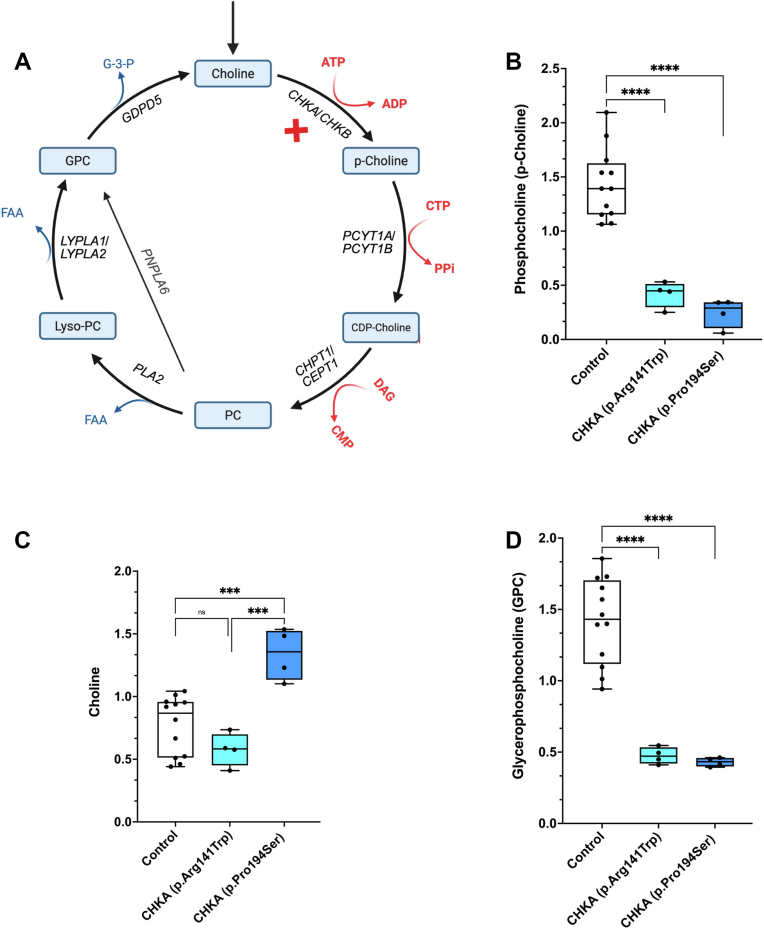
**Targeted metabolic profiling of *CHKA* patient fibroblasts**. *A*, schematic presentation of the CDP-choline branch of the Kennedy pathway. Levels of p-choline (*B*), choline (*C*), and GPC-choline (*D*) in skin fibroblasts from controls and patients carrying biallelic *CHKA* variants (p.Pro194Ser or p.Arg141Trp). Control data were obtained from skin fibroblasts isolated from three healthy individuals. Data are shown as individual points representing technical replicates from three independent experiments. Statistical significance was determined by one-Way ANOVA followed by Dunnett’s test for multiple comparisons; ∗∗∗*p* < 0.001, ∗∗∗∗*p* < 0.0001.

Rare, biallelic recessive variants in *CHKA* cause a neurodevelopmental disorder characterized by epilepsy and microcephaly (OMIM #620023) classified as a neurodevelopmental form of complex spastic paraplegia (17). Amongst these patients, we identified three missense variants, one start-loss variant, and one truncating variant (17). Missense variants substantially reduced enzyme activity, while start-loss and truncations are predicted to eliminate activity. In contrast to *CHKA* disease-causing variants, autosomal recessive variants in *CHKB* cause a neuromuscular disorder characterized by muscular dystrophy with intellectual disability and cardiomyopathy (OMIM #602541) (20, 21, 50, 51, 52). How variants in two genes that catalyze the same biochemical reaction for the synthesis of PC cause inherited diseases with different phenotypes is unclear, with differences in expression pattern, substrate affinity, or complementation for loss of function by one choline kinase isoform by increased expression of the other isoform being potential contributors (4, 17, 20, 21, 50, 51, 53, 54, 55, 56).

In this study, we investigated biochemical and cellular alterations due to known disease-causing biallelic *CHKA* variants in patient-derived fibroblasts. The main aims were to increase knowledge of disease etiology and identify potential processes that could be targeted for therapeutic intervention.

## Results

### Biallelic CHKA variants alter cellular lipid and metabolite profiles

The levels of the metabolites in the Kennedy pathway for PC synthesis were determined by targeted metabolomic mass spectrometry in normal fibroblasts and patient-derived fibroblasts carrying known homozygous *CHKA* disease causing variants (p.Arg141Trp and p.Pro194Ser). Previous work determined that the p.Arg141Trp variant retained 25% enzymatic activity while the p.Pro194Ser variant possessed 17% activity (17). Metabolomic analysis revealed that the mass of phosphocholine (p-choline), the product of choline kinase, was decreased to 28% of control values in p.Arg141Trp fibroblasts and to 17% in p.Pro194Ser fibroblasts (Fig. 1*B*), consistent with the reported decrease in choline kinase activity (17). Choline, the substrate of this reaction, was increased to 130% of control in the p.Pro194Ser fibroblasts but statistically unchanged in the p.Arg141Trp cells (Fig. 1*C*). The increase in choline substrate in p.Pro194Ser fibroblasts is consistent with prior evidence indicating a greater impairment in choline kinase activity associated with this variant (17). When PC synthesis is impaired the production of its catabolic degradation product glycerophosphocholine (GPC) is often decreased (Fig. 1*A*) (57, 58, 59, 60, 61, 62). Consistent with this, the level of GPC in both patient-derived fibroblast cell lines was 30% of control values (Fig. 1*D*). The metabolite profiles illustrate that loss-of-function CHKA alleles result in reduced overall choline kinase activity in the Kennedy pathway and impair PC turnover.

**Figure 2:**
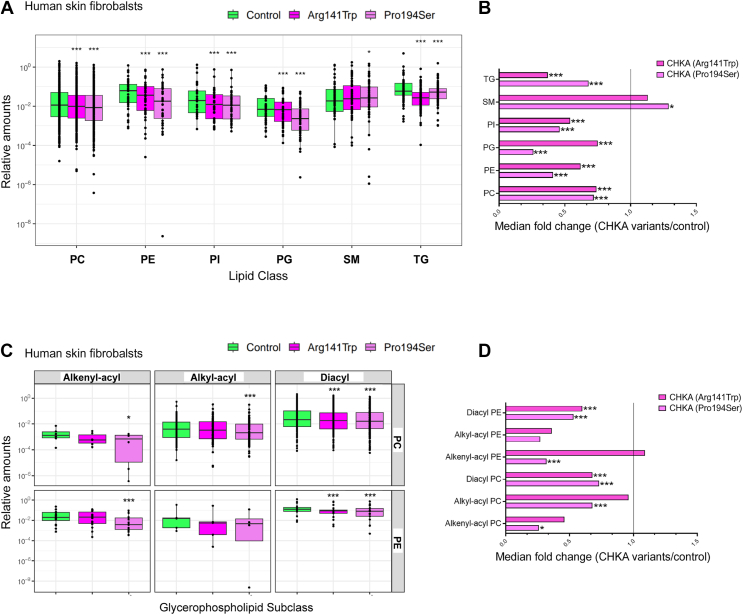
**Biallelic *CHKA* variants are associated with altered lipid profile**. Expression levels of major lipids (*A*) and summary of fold change in major lipid species (*B*), as well as PC and PE subspecies (*C*) and their median fold change vs control (*D*) in patient fibroblasts carrying biallelic *CHKA* variants (p.Pro194Ser or p.Arg141Trp). Control data were obtained from skin fibroblasts isolated from three healthy individuals. The bounds of the boxplots correspond to the 25th and 75th percentiles of data with the center line corresponding to the median value and each dot representing an individual lipid species from three independent experiments. The *upper*/*lower* whiskers extend from the hinges to the largest/smallest value no more than 1.5 times the interquartile range away from the hinges (*A* and *C*). The significance of a median pair-wise fold-change in lipid amounts was determined using Pairwise Wilcoxon signed rank test with Bonferroni correction; ∗*p* < 0.05, ∗∗∗*p* < 0.001. PC, Phosphatidylcholine; PE, phosphatidylethanolamine, PI, phosphatidylinositol, PG, phosphatidylglycerol, SM, sphingomyelin, TG, triacylglycerol.

Next, the impact of biallelic *CHKA* variants on fibroblast phospholipid levels was determined by lipidomic profiling. Patient-derived fibroblasts had a marked reduction in major lipid classes. PC was reduced by 75% compared to controls including decreased alkyl-acyl and alkenyl-acyl linked PC. Phosphatidylethanolamine, phosphatidylinositol, phosphatidylglycerol, and triacylglycerol levels were also reduced by 30 to 70% relative to control (Fig. 2, *A*–*D*). Importantly, the decrease in PC mass and the differences in PC Kennedy pathway metabolites are consistent with the patient-derived *CHKA* alleles causing decreased PC synthesis.

### Biallelic CHKA variants are associated with increased nuclear CCTα translocation

**Figure 3:**
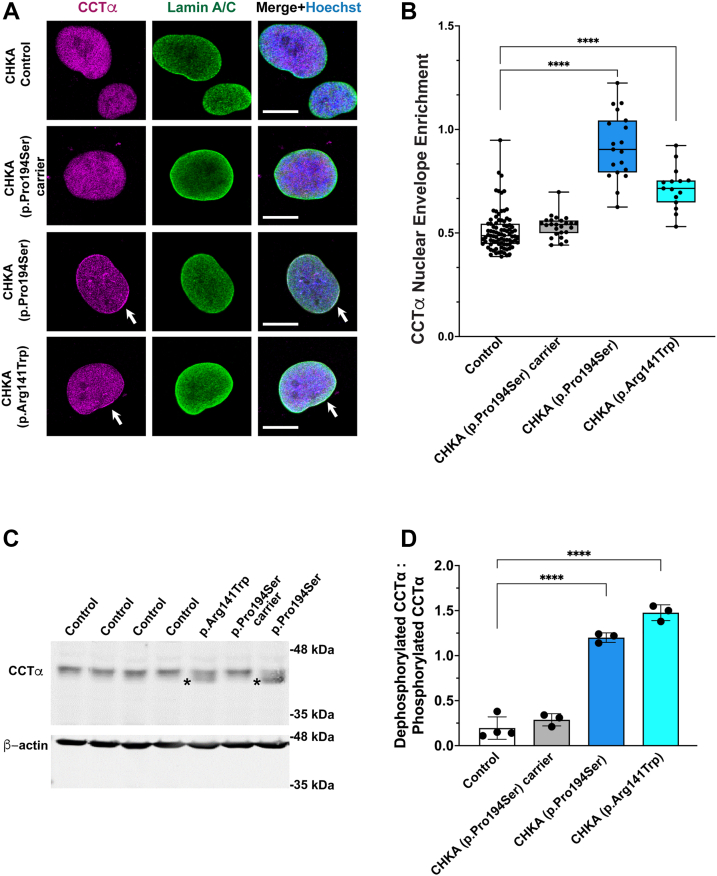
**Biallelic *CHKA* variants are associated with increased nuclear envelope translocation of CCTα**. *A*, representative images of patient-derived fibroblasts carrying biallelic CHKA variants (p.Pro194Ser or p.Arg141Trp) and fibroblasts heterozygous for the p.Pro194Ser variant, co-immunostained with antibodies against PCYT1A (CCTα) and lamin A/C. Control fibroblasts were derived from skin biopsies of three healthy individuals. *B*, quantification of CCTα enrichment at the nuclear envelope. Data represent the mean ± SD from 15 to 26 nuclei per condition, collected from three independent experiments. Statistical significance was determined by one-way ANOVA followed by Dunnett’s *post hoc* test; ∗∗∗∗*p* < 0.0001. *C*, Western blot analysis of CCTα expression in control and CHKA variant fibroblasts; β-actin was used as a loading control. *D*, quantification of the ratio of the lower to upper CCTα bands, reflecting the relative abundance of dephosphorylated *versus* phosphorylated forms (39, 65). Bars represent mean ± SD from three independent experiments. Statistical significance was determined by one-way ANOVA followed by Dunnett’s *post hoc* test; ∗∗∗∗*p* < 0.0001.

As CHKA catalyzes the first step of the CDP–choline pathway, its deficiency leads to reduced phosphocholine production and consequently decreased phosphatidylcholine (PC) synthesis. Previous studies have shown that reduced PC levels activate PCYT1A (CCTα), the major and ubiquitous CTP:phosphocholine cytidylyltransferase isoform that catalyzes the rate-limiting step for PC synthesis, by promoting its translocation from the nucleoplasm to the inner nuclear membrane (25, 26, 27, 29, 32, 33, 34, 63, 64, 65). The inactive, soluble form of PCYT1A (CCTα) is localized to the nucleoplasm but becomes activated upon translocation to the inner nuclear membrane, where conformational changes increase its enzymatic activity (25, 26, 27, 29, 32, 33, 34, 63, 65, 66, 67, 68, 69). Membrane binding by CCTα is mediated by a C-terminal amphipathic helix that associates with membranes enriched in anionic lipids (*e*.*g*., PA, PS) or non-bilayer lipids (*e*.*g*., PE, DAG) that induce packing defects and membrane curvature stress (25, 26, 29, 32). To determine whether a similar compensatory mechanism occurs in CHKA-deficient cells, we examined the subcellular localization and activation state of CCTα in fibroblasts carrying biallelic CHKA variants. Immunofluorescence microscopy using antibodies against CCTα and the nuclear envelope protein lamin A/C revealed increased CCTα immunostaining at the nuclear envelope in *CHKA* variant fibroblasts compared to controls (Fig. 3*A*), with a significant increase in the nuclear envelope (NE) enrichment index (ratio of NE to nucleoplasmic staining) (Fig. 3*B*). Fibroblasts heterozygous for the p.Pro194Ser variant showed a pattern similar to control cells (Fig. 3, *A* and *B*).

Consistent with the increased NE enrichment of CCTα in CHKA variant fibroblasts, Western blot analysis indicated that CCTα was predominantly in its dephosphorylated (active) form (27, 39, 64, 65), as shown by the lower molecular mass band (Fig. 3*C*, asterisks). In contrast, CCTα in control and carrier fibroblasts appeared as a slower migrating band, corresponding to its inactive form due to extensive phosphorylation of the C-terminal P-domain (27, 64, 65).

Together, these data suggest that loss of CHKA activity results in a compensatory activation of CCTα at the inner nuclear membrane, likely as an adaptive response to restore PC synthesis.

### Biallelic *CHKA* variants are associated with altered expression of key regulators of lipid metabolism

**Figure 4:**
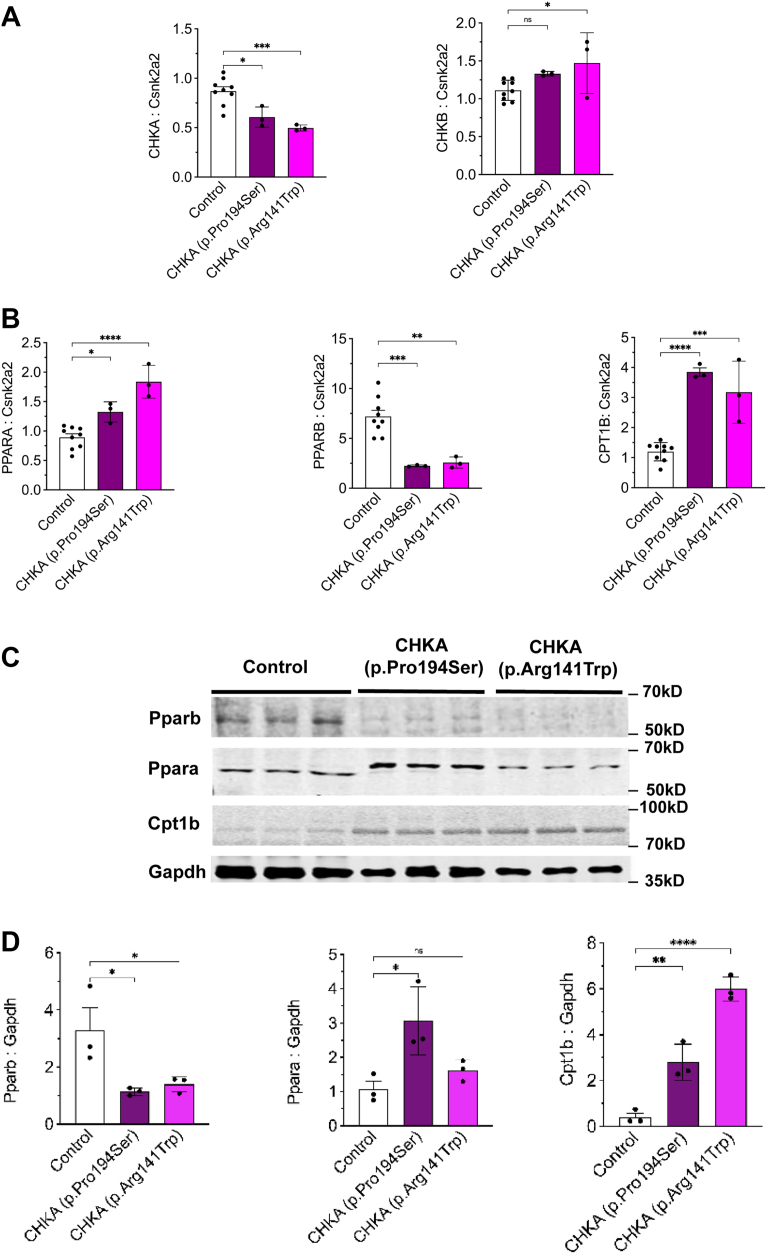
**Biallelic *CHKA* variants (p.Pro194Ser or P.Arg141Trp) are associated with altered expression of key lipid metabolism regulators**. *A–B*, quantitative PCR analysis of *CHKA*, *CHKB*, *PPARA*, *PPARB* and *CPT1B* mRNA levels in fibroblasts carrying biallelic CHKA variants. Control is the mean of three different normal human skin fibroblast lines. *C*, Western blot analysis of PPARA, PPARB, and CPT1B protein expression. *D*, quantification of PPARA, PPARB, and CPT1B western blots relative to GAPDH. Results are the mean ± SD from three independent experiments. Statistical significance was determined by one-way ANOVA followed by Tukey’s test for multiple comparisons; ∗*p* < 0.05, ∗∗*p* < 0.01, ∗∗∗∗*p* < 0.0001.

Our investigation into the impact of biallelic *CHKA* variants on lipid regulatory pathways was informed by previous findings in *Chkb* knockout mice (13, 20, 21, 51, 70, 71). *Chkb*^*−/−*^ mice present with a rostrocaudal muscular dystrophy, where affected muscle displayed a decrease in *Chka* isoform expression, while unaffected muscle displayed an increase in *Chka* expression (13, 20, 21, 51). The previous observation that AAV-mediated expression of human CHKA in *Chkb*^*−/−*^ mice ameliorated the muscular dystrophy phenotype (72) suggests that increased CHKA expression could compensate for loss of CHKB function. Thus, we used quantitative PCR to determine if there were changes in *CHKB* expression in the patient-derived *CHKA* fibroblasts. The expression of *CHKA* was reduced to 50% of control level but only a small increase in *CHKB* expression was observed in one of the patient-derived *CHKA* fibroblasts (Fig. 4*A*) suggesting no compensatory role for *CHKB*.

In addition to changes in choline kinase isoform expression in the *Chkb*^*−/−*^ mice, the expression of peroxisome proliferator activated receptors (PPARs) and their downstream target genes was also decreased (21). In *Chkb*^*−/−*^ mouse myocytes, treatment with PPAR agonists prevented the observed lipid metabolic defects and resulted in upregulation of the *Chka* isoform, preventing cell injury (21). Given this precedent, we assessed the expression of PPAR family members and associated metabolic genes in *CHKA* patient-derived fibroblasts. Expression of *PPARB* mRNA was significantly downregulated in both fibroblast lines, while *PPARA* was significantly upregulated in fibroblasts carrying the p.Pro194Ser and p.Arg141Trp variants (Fig. 4*B*). In addition, *CPT1B*, a key enzyme mediating mitochondrial fatty acid import into the mitochondria for β-oxidation, was significantly upregulated (Fig. 4*B*). These changes in mRNA expression resulted in corresponding effects on protein expression determined by Western blot analysis (Fig. 4, *C* and *D*), supporting a shift in PPAR regulation of lipid metabolism and a parallel, yet distinct, regulatory response to *CHKA* deficiency compared to *Chkb* loss-of-function models.

### Defective mitochondrial function in patient fibroblasts with biallelic CHKA variants

**Figure 5:**
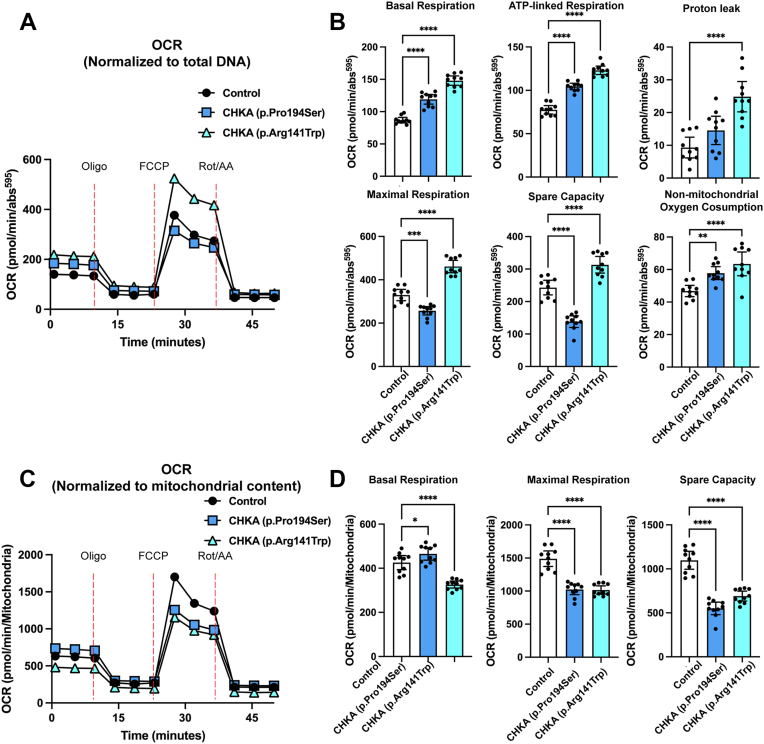
**Analysis of mitochondrial and glycolytic functions in patient fibroblasts carrying biallelic *CHKA* variants**. Oxygen consumption rate (OCR) in fibroblasts carrying biallelic *CHKA* variants and controls (mean of three different normal human skin fibroblast lines) normalized to DNA content (*A–B*) or mitochondrial content (*C–D*). Sequential injections of oligomycin (1 μM), FCCP (1 μM), and a mixture of rotenone (1 μM) and antimycin A (1 μM) were used to assess mitochondrial respiration, respectively. Data are shown as individual points representing technical replicates (individual wells) from three independent experiments; bars represent mean ± SD. Statistical significance was determined by one-way ANOVA followed by Dunnett’s test for multiple comparisons; ∗*p* < 0.001, ∗∗*p* < 0.001, ∗∗∗*p* < 0.001, ∗∗∗∗*p* < 0.0001.

Given the observed alterations in key lipid metabolic and β-oxidation regulators including PPARs and CPT1B, we next examined the impact of biallelic *CHKA* variants on mitochondrial bioenergetics using Seahorse extracellular flux analysis. Mitochondrial respiration was assessed and normalized to total DNA content. Both patient-derived fibroblast lines exhibited elevated basal respiration, with increases in ATP-linked and non-mitochondrial respiration. Proton leak was significantly elevated in fibroblasts carrying the p.Arg141Trp variant (Fig. 5, *A* and *B*). Assessment of maximal respiratory capacity revealed that the p.Arg141Trp variant showed significantly increased maximal respiration and spare capacity, whereas the p.Pro194Ser variant exhibited significant reductions in both parameters (Fig. 5, *A* and *B*). However, when respiration was normalized to mitochondrial content basal respiration was reduced in both *CHKA* variant lines (Fig. 5, *C* and *D*). Similarly, maximal respiration and spare capacity were reduced in cells carrying either variant, suggesting compromised mitochondrial efficiency per organelle.

### Biallelic CHKA patient-derived variants are associated with increased mitochondrial fragmentation, ROS production, and lipid peroxidation

**Figure 6:**
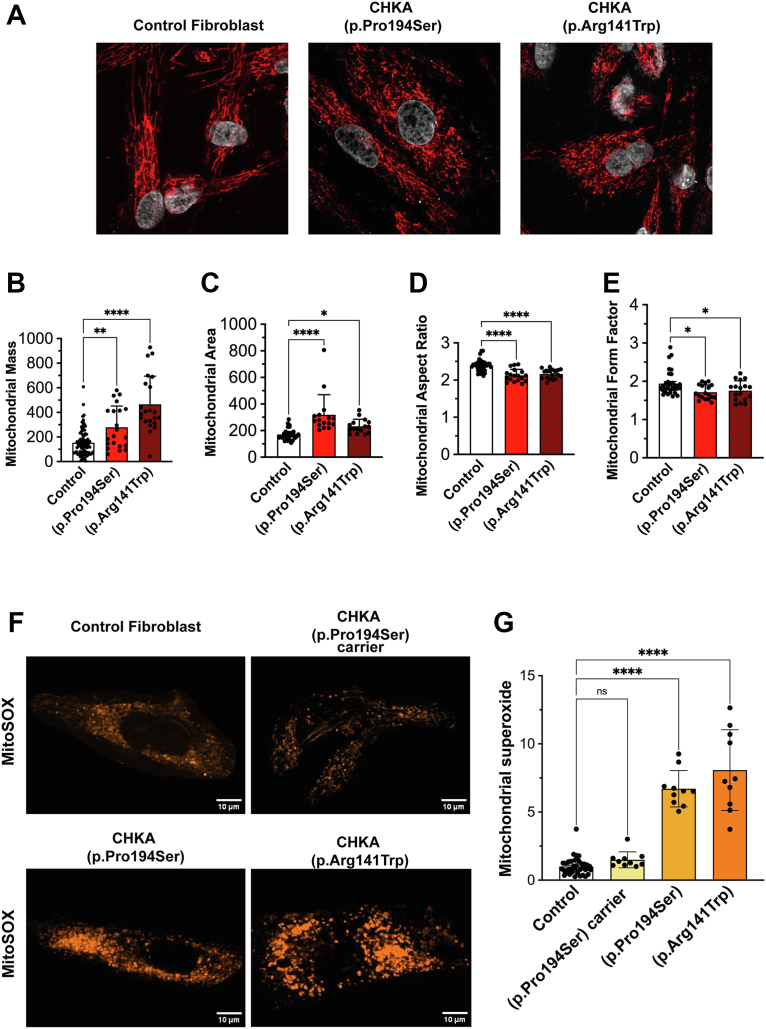
**Biallelic *CHKA* variants (p.Pro194Ser or P.Arg141Trp) are associated with increased mitochondrial fragmentation and ROS production**. *A*, representative images of fibroblasts carrying biallelic *CHKA* variants (p.Pro194Ser or p.Arg141Trp) and with controls (mean of three different normal human skin fibroblast lines), stained with MitoTracker Deep Red to visualize mitochondrial morphology. *B–E*, Confocal z-stack images of patient fibroblasts were processed to calculate mitochondrial mass, area, aspect ratio, and form factor using ImageJ 1.54p software. Data are shown as individual points representing separate image fields from three independent experiments; bars represent mean ± SD. Statistical significance was determined by one-way ANOVA followed by Dunnett’s test for multiple comparisons; ∗*p* < 0.05, ∗∗*p* < 0.01, ∗∗∗∗*p* < 0.0001. *F*, representative images of fibroblasts carrying biallelic *CHKA* variants (p.Pro194Ser and p.Arg141Trp) and fibroblasts heterozygous for the p.Pro194Ser variant stained with MitoSOX to measure mitochondrial ROS production. *G*, quantification of MitoSOX fluorescence intensity normalized to cell number. Data are shown as individual points representing separate image fields from three independent experiments; bars represent mean ± SD. Statistical significance was determined using one-way ANOVA followed by Dunnett’s test for multiple comparisons; ∗∗∗∗*p* < 0.0001.

Given the observed increase in basal mitochondrial respiration, yet impaired mitochondrial respiratory efficiency, we evaluated mitochondrial morphology and reactive oxygen species (ROS) production in biallelic *CHKA* variant fibroblasts. To quantitatively assess mitochondrial network organization, we measured the aspect ratio and form factor of individual mitochondria. The aspect ratio represents the ratio of the major to minor axis of each mitochondrion, providing an estimate of elongation, with higher values indicate more elongated mitochondria, while lower values reflect rounder or fragmented structures (73). The form factor incorporates both length and branching complexity, calculated from mitochondrial area and perimeter; higher values denote more interconnected, tubular networks, whereas lower values indicate a fragmented or punctate morphology (73). Both patient fibroblast lines exhibited increased mitochondrial mass and area, suggestive of mitochondrial remodeling. This was accompanied by a significant reduction in aspect ratio and form factor, consistent with a shift toward a fragmented mitochondrial phenotype (Fig. 6, *A*–*E*).

**Figure 7:**
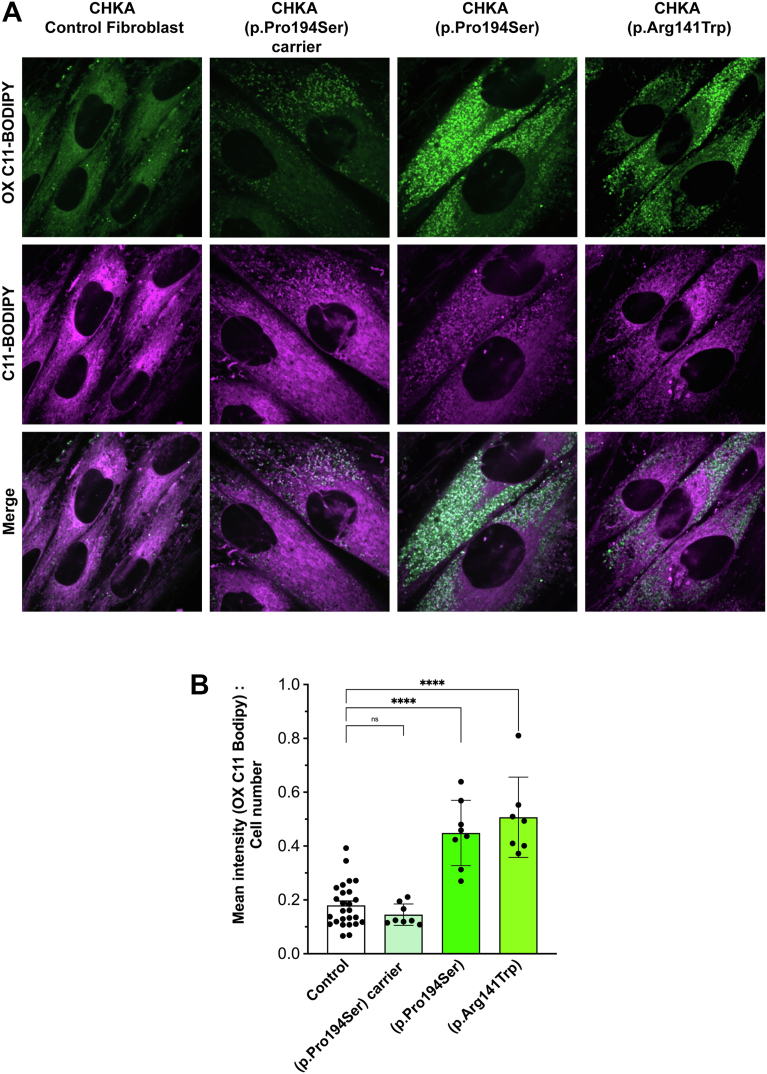
**Biallelic *CHKA* variants (p.Pro194Ser or P.Arg141Trp) are associated with increased lipid peroxidation**. *A*, representative images of fibroblasts carrying biallelic *CHKA* variants (p.Pro194Ser or p.Arg141Trp), fibroblasts heterozygous for the p.Pro194Ser variant and controls (mean of three different normal human skin fibroblast lines), stained with C11-BODIPY to determine lipid peroxidation. *B*, quantification of oxidized C11- BODIPY fluorescence intensity to cell number ratio. Data are shown as individual points representing separate image fields from three independent experiments; Bars represent mean ± SD. Statistical significance was determined using one-way ANOVA followed by Dunnett’s test for multiple comparisons; ∗*p* < 0.05, ∗∗∗∗*p* < 0.0001.

ROS levels were significantly elevated in fibroblasts carrying biallelic *CHKA* variants. In contrast, non-pathogenic fibroblasts heterozygous for the p.Pro194Ser variant exhibited ROS levels comparable to controls, reinforcing the conclusion that the mitochondrial defects and oxidative stress phenotypes are specifically associated with a substantial decrease in *CHKA* activity (Fig. 6, *F* and *G*). Given the observation of increased ROS levels in patient fibroblasts harboring biallelic *CHKA* variants, we sought to determine whether these variants caused increased lipid peroxidation using the lipid peroxidation sensor C11-BODIPY. Indeed, C11-BODIPY staining shifted from red to green signifying higher lipid peroxidation in the fibroblasts from patients with the biallelic *CHKA* variants. In contrast, no significant increase in lipid peroxidation was observed in fibroblasts carrying the heterozygous p.Pro149Ser variant or controls (Fig. 7, *A* and *B*).

We next sought to investigate whether increased lipid peroxidation observed in patient fibroblasts harboring the biallelic *CHKA* variants is directly due to impaired CHKA activity. We treated U2OS and SH-SY5Y cells with EB-3D, a potent and selective CHKA inhibitor (23, 74, 75), and assessed lipid peroxidation levels. Our results show increased lipid peroxidation in a dose-dependent manner as evident by increased ratio of oxidized to reduced C11-BODIPY (Fig. S1). Together with results from patient fibroblasts, these findings support the hypothesis that impaired CHKA activity increases lipid peroxidation.

### FCCP reduces ROS production and lipid peroxidation in CHKA variants

**Figure 8:**
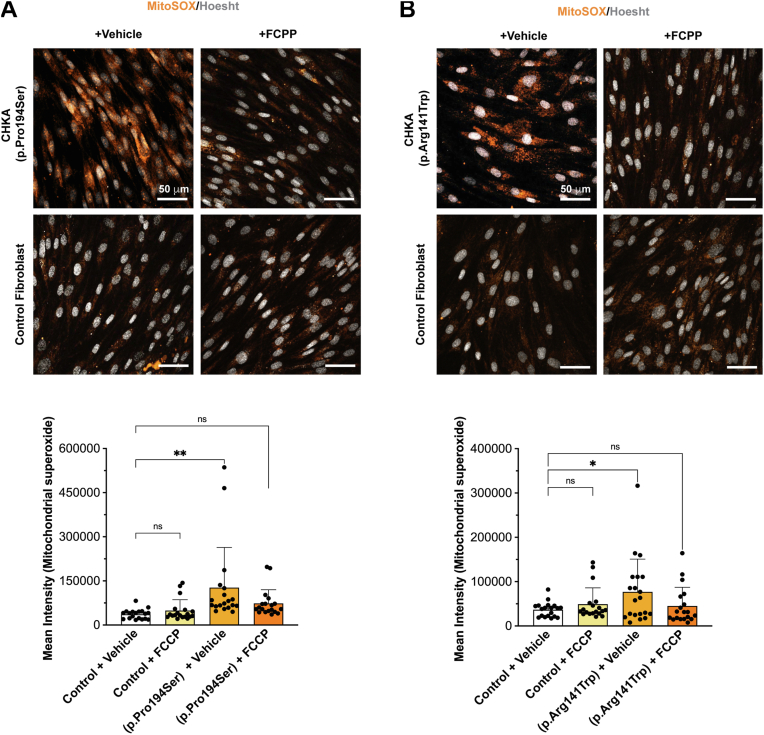
**FCCP reverses the increase in ROS production in fibroblasts carrying biallelic *CHKA* variants**. *A–B*, representative images of fibroblasts carrying biallelic CHKA variants (p.Pro194Ser or p.Arg141Trp) and control fibroblasts derived from three independent healthy skin fibroblast lines, stained with MitoSOX to assess ROS production following 72 h of FCCP treatment. Quantification of MitoSOX mean fluorescence intensity is shown below. Data are presented as individual points representing independent image fields from three independent experiments; bars represent mean ± SD. Statistical significance was determined by one-way ANOVA followed by Dunnett’s *post hoc* test for multiple comparisons; ∗*p* < 0.05, ∗∗*p* < 0.01, ∗∗∗*p* < 0.001, ∗∗∗∗*p* < 0.0001.

**Figure 9:**
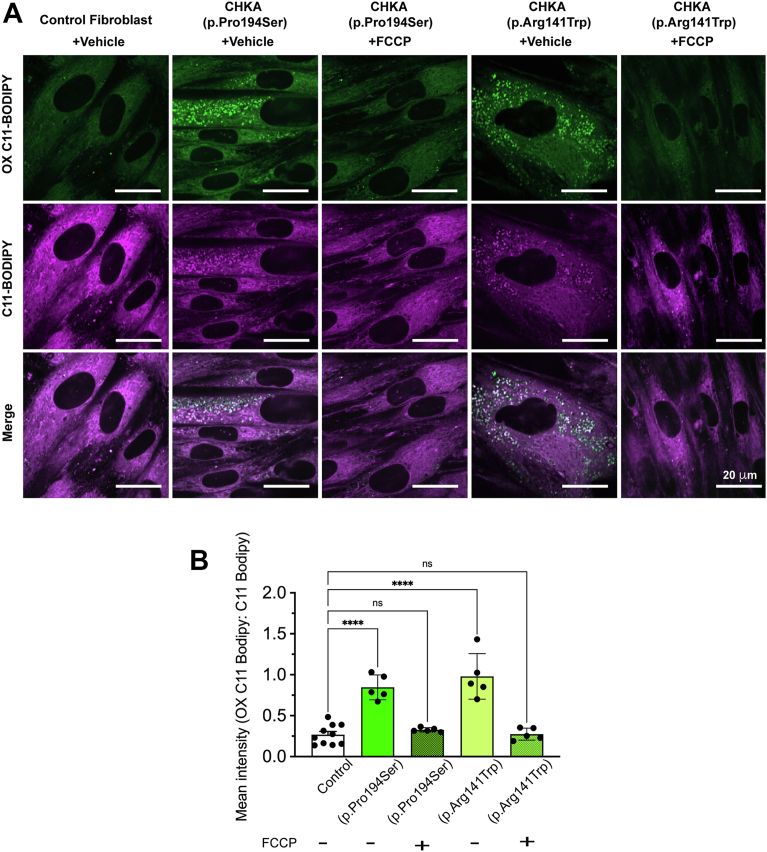
**FCCP reverses the increase in lipid peroxidation observed in fibroblasts carrying biallelic *CHKA* variants**. *A*, Representative images of fibroblasts carrying biallelic *CHKA* variants (p.Pro194Ser or p.Arg141Trp) stained with C11-BODIPY to measure lipid peroxidation levels after treatment with FCCP for 72 h. *B*, quantification of oxidized to reduced C11-BODIPY fluorescence intensity ratio. Data are shown as individual points representing separate image fields from three independent experiments; bars represent mean ± SD. Statistical significance was determined using one-Way ANOVA followed by Dunnett’s test for multiple comparisons; ∗∗∗∗*p* < 0.0001.

Given the observed increase in ROS and lipid peroxidation in fibroblasts carrying the *CHKA* variants, we next investigated whether pharmacological modulation of mitochondrial function with FCCP, a mitochondrial uncoupler known to decrease free radical production (76, 77, 78), could mitigate the observed oxidative stress and lipid peroxidation. After treatment with FCCP, patient fibroblasts harboring the biallelic *CHKA* variants exhibited a reduction in ROS levels to near those of control cells (Fig. 8, *A* and *B*). Correspondingly, the reduction in ROS levels upon FCCP treatment was accompanied by a reduction in lipid peroxidation, as demonstrated by C11-BODIPY staining, to levels similar to control cells (Fig. 9, *A* and *B*). These findings suggest that mitochondrial uncoupling with FCCP alleviates the lipid oxidative stress-related phenotype associated with biallelic *CHKA* variants.

## Discussion

This study demonstrates that biallelic pathogenic variants in *CHKA* lead to a global reduction in phospholipids including PC and its biosynthetic intermediates that negatively impact mitochondrial function, increase oxidative stress, and elevate ROS and lipid peroxidation in patient-derived fibroblasts. Importantly, treatment of patient-derived *CHKA* deficient fibroblasts with a mitochondrial uncoupler ameliorated ROS production and lipid peroxidation suggesting an important mechanism by which lipids are damaged when CHKA activity is defective.

Metabolomics identified a decrease in the CHKA product p-choline, PC (the major product of the Kennedy pathway), and the downstream PC catabolite GPC, consistent with a severe reduction in PC synthesis. The pathogenic variants studied here retain approximately 17 to 25% of wild-type choline kinase enzymatic activity (17). To date, no patients with a complete loss of CHKA-encoded enzymatic activity have been described (17). This is consistent with the observation that *Chka* knockout in mice is embryonic lethal (79), while mice heterozygous for *Chka* displayed similar brain phenotypes to patients with *CHKA*, including abnormal white matter and subcortical and connectivity regions, commissural defects and microcephaly (80).

Interestingly, decreased CHKA enzymatic activity in patient-derived fibroblasts was not accompanied by a compensatory increase in CHKA mRNA expression. In addition, no major changes in CHKB expression were observed in these cell lines. These findings indicate that (i) there is no feedback mechanism to upregulate CHKA or CHKB expression in response to reduced CHKA activity, and (ii) CHKB activity alone is insufficient to sustain normal phosphatidylcholine (PC) synthesis or prevent the cellular phenotypic defects caused by CHKA deficiency. This contrasts the *Chkb*^*−/−*^ mouse model of *CHKB* mediated rostrocaudal muscular dystrophy where (1) unaffected muscle displayed an increase in expression of *CHKA* and was spared from disease (21, 50, 51, 70, 71) and (2) ectopic expression of *CHKA* in *Chkb*^*−/−*^ mouse muscle was sufficient to restore normal muscle structure and function (72). It is clear from this and past studies that *CHKA* and *CHKB* are not functionally equivalent even though they catalyze the same enzymatic reaction.

This is the first in-depth study of lipidomic alterations due to defective CHKA activity. In fibroblasts carrying biallelic *CHKA* variants, lipidomic analysis revealed a marked reduction in PC along with most major phospholipids as well as the neutral storage lipid TG. Similar lipid alterations have been documented in other cellular models of phospholipid biosynthesis disruption, such as CHO MT58 cells with a temperature-sensitive mutation in *PCYT1* (CCTα) (81). Fibroblasts carrying biallelic *CHKA* variants had increased nuclear membrane-associated and active dephosphorylated PCYT1 (CCTα), a compensatory response to decreased CHKA activity that was not sufficient to restore PC to normal levels based on our metabolomic and lipidomic results. A similar PCYT1 (CCTα) compensatory response was reported in cells with a knockout of *CEPT1* and *CHPT1* that reduced PC synthesis and increased CCTα nuclear membrane enrichment and elevated expression of its active dephosphorylated form (39). The mechanisms by which altered PC levels influence other lipid species are likely driven by extensive ROS damage to membranes and the inter-regulation of lipid metabolic pathways that are dependent on integral membrane bound enzymes and their interactions with membrane lipids for optimal activity (3, 5, 6, 14, 20, 21, 26, 51, 56, 63, 66, 82, 83, 84). In addition, PC is the predominant bilayer-forming lipid in cells and its reduction may alter the activity of numerous integral and peripheral membrane proteins, many of which are directly involved in lipid synthesis. These possibilities, alone or in combination, warrant further investigation.

Loss of function of *CKHB* in patients and *Chkb*^*−/−*^ mice resulted in increased mitochondrial size and defective mitochondrial respiration (21, 50, 51, 85). Our investigation into mitochondrial function in patient-derived *CHKA* fibroblasts revealed increased basal and maximal respiration and spare capacity in both *CHKA* variant lines, which is a compensatory response to mitochondrial dysfunction that is manifested due to increased mitochondrial mass per cell. However, when respiration was normalized to mitochondrial content, basal, maximal and spare capacity respiration were reduced, indicating compromised mitochondrial efficiency. These findings align with our observation of elevated ROS and lipid peroxidation in fibroblasts carrying the biallelic *CHKA* variants. The association between impaired CHKA activity and elevated lipid peroxidation is further supported by our findings in U2OS bone osteosarcoma cells and SH-SY5Y neuroblastoma cells treated with the CHKA inhibitor EB-3D, which demonstrated a dose-dependent increase in lipid peroxidation (Fig. S1). Previous studies showed that lipid peroxidation adversely affects synaptic function and neuronal survival *in vitro*. Although mitochondrial dysfunction is commonly reported in many known inherited diseases of the Kennedy pathway (4, 21, 46, 56, 85), how defects in PC synthesis affect the overall lipidome at the organellar level, and how these changes contribute to impaired mitochondrial function remains understudied and warrants further investigation. To determine if the increase in mitochondrial-generated ROS was causing lipid peroxidation, we explored the potential of FCCP, a mitochondrial uncoupler (78, 86, 87), to mitigate the oxidative stress and lipid peroxidation observed in *CHKA* patient fibroblasts. FCCP treatment significantly reduced ROS levels and lipid peroxidation to a level similar to fibroblasts from controls. While it is uncertain whether mitochondrial uncoupling is sufficient to restore phospholipid and TG levels in *CHKA* variant fibroblasts, this initial observation implies that mitochondrial uncoupling could be a promising approach to alleviate pathological cellular defects associated with defective *CHKA* variants.

## Experimental procedures

### Cell culture

Skin fibroblasts were collected from *CHKA* affected individuals and control fibroblasts derived from three independent healthy skin fibroblast lines. Cells were cultured in Dulbecco's Modified Eagle's Medium (DMEM) supplemented with 10% fetal bovine serum (Gibco) and 1% Antibiotic-Antimycotic (AA, Gibco). Cells were maintained at 37 °C in 5% CO_2_ (v/v) and cells below passage 15 were used in experiments.

### Lipidomics

Skin fibroblasts were seeded at a density of 1 × 10^6^ cells per 10 cm dish in complete DMEM and incubated overnight at 37 °C in 5% CO_2_. Lipids were extracted using a modified Bligh and Dyer method for LC-MS analysis as previously described (21, 51). Lipid analysis was performed using UHPLC coupled with high-resolution tandem mass spectrometry, and lipid identification and quantification were conducted using the Thermo Scientific LipidSearch software version 4.2 as described previously (21, 43).

### Metabolomics

Patient fibroblasts were seeded at a density of 1 × 10^6^ cells per 10 cm dish in complete DMEM and maintained overnight at 37 °C with 5% CO_2_. The next day, cells were harvested in 200 μl of distilled water and lysed by probe sonication (2 cycles, power setting 1; Fisher Sonic Dismembrator 100). Targeted metabolomic profiling was carried out by high-performance liquid chromatography coupled to a linear ion trap triple-quadrupole tandem mass spectrometer (LC-MS/MS) (21). Peak areas were quantified using Skyline (version 21.0), an open-source MRM analysis platform. Data quality control, normalization, and visualization were performed with custom R scripts (version 4.0.2). For each metabolite, sample peak intensities were normalized to the mean intensity of the two nearest flanking QC pool samples (generated by combining all study samples) to correct for signal drift.

### CCTα nuclear membrane localization

Fibroblasts cultured on glass coverslips for 48 h were fixed with 4% paraformaldehyde for 15 min, washed twice with 5 mM ammonium chloride in PBS, and permeabilized with 0.2% Triton X-100 in PBS at 4 °C for 10 min. Cells were then blocked with 1% (w/v) bovine serum albumin (BSA) in PBS (blocking buffer) for 60 min, followed by staining with anti-PCYT1 (CCTα) antibody (polyclonal antibody raised in rabbit against a peptide from the C-terminal phosphorylation domain; GenScript) (88) and anti-LMN A/C (Cell Signaling; cat: #4C11) in blocking buffer overnight at 4 °C. Primary antibody was removed and coverslips were washed twice with blocking buffer followed incubations with secondary goat anti-rabbit AlexaFluor555 and goat anti-mouse AlexaFluor647 for 60 min in blocking buffer. Secondary antibody was removed, and fibroblasts were stained with Hoechst (1 μg/ml) for 7 min in PBS followed by two additional PBS washes. Coverslips were then mounted onto glass slides with Mowiol 4 to 88 (Calbiochem). Coverslips were imaged using a Leica SP8 confocal microscope at 63x objective magnification with an HC Plan APOCHROMAT CS2 lens with a numerical aperture of 1.4 using the Lightning setting (2–3 fields of cells were imaged for each fibroblast line).

Quantification of nuclear envelope (NE) PCYT1 enrichment in fibroblasts was performed using ImageJ, as originally described by Lee *et al*. (2023) (89). The nuclei from raw confocal images were cropped and then converted to 8 bit grey-scale for each channel, with the signal of the Hoechst and PCYT1 channels being added together and converted to a binary nuclear mask, which was used to select the entire nucleus in the PCYT1 channel and measure the mean intensity values of the total nucleus. The selection was then adjusted by −10 pixels to select the nucleoplasm in the PCYT1 channel and the mean intensity was measured once more. Finally, the nucleoplasm selection was applied to the total nuclear mask to subtract the nucleoplasmic region and leave only the nuclear envelope region. This mask was used as a selection to measure the mean intensity of PCYT1 at the nuclear envelope (NE). The mean intensity PCYT1 at the NE was divided by the mean intensity of nucleoplasmic CCTα to calculate the NE enrichment score. For each fibroblast cell line, 15 to 26 cell nuclei were analyzed from 2 to 3 fields of cells.

### Mitochondrial ROS production

Mitochondrial superoxide production was quantified using MitoSOX Red reagent (Invitrogen). Skin fibroblasts (4 × 10^4^) were seeded onto an 8-well chamber slide (Ibidi) and incubated overnight at 37 °C in 5% CO_2_. Cells were incubated with MitoSOX (1 μM) and Hoechst (2 μM) for 30 min at 37 °C in serum-free DMEM at 37 °C, 5% (*v/v*) CO_2_. Following incubation, cells were washed twice with PBS to remove any excess dye and FluoroBrite DMEM (Gibco) was added prior to imaging. Images were acquired using a Zeiss Cell Observer Spinning-Disk microscope with an excitation wavelength of 551 nm for MitoSOX and 353 nm for Hoechst. MitoSOX signal was quantified using Fiji (ImageJ) software. A threshold was applied to acquired images to subtract background fluorescence. Mitochondrial superoxide levels were then quantified by measuring the integrated density, which reflects the total fluorescence within a defined region of interest. This value was normalized to cell number to calculate mean fluorescence intensity.

### Quantification of mitochondrial morphology

Mitochondrial morphology was assessed using MitoTracker Deep Red (Thermo Fisher Scientific). Fibroblasts were seeded onto an 8-well chamber slide (Ibidi) at a density of 4 × 10^4^ and incubated overnight at 37 °C, 5% (v/v) CO_2_. Cells were incubated with Mitotracker Deep Red (200 nM) in serum-free DMEM at 37 °C, 5% (*v/v*) CO_2_ for 30 min. Following incubation, cells were washed twice with PBS to remove excess dye, and FluoroBrite DMEM (Gibco) was added prior to imaging. Images were then acquired using a Zeiss Cell Observer Spinning-Disk microscope with an excitation wavelength of 641 nm. The acquired images were processed using ImageJ 1.54p software to quantify mitochondrial morphological parameters. Image processing included the following sequential steps (1): Z-projection (2), background subtraction (3), local contrast enhancement (4), gamma correction (5), thresholding (6), despeckling, and (7) removal of outliers, as previously described by Chaudhry *et al*. (2020) (73).

### Quantification of lipid peroxidation levels

Lipid peroxidation levels were assessed using the lipid peroxidation sensor C11-BODIPY (Thermo Fisher Scientific). Fibroblasts (4 × 10^4^) were seeded onto an 8-well chamber slide (Ibidi) and incubated overnight at 37 °C and 5% CO_2_. Cells were incubated with C11-BODIPY (2 μM) in serum-free DMEM at 37 °C and 5% CO_2_ for 20 min. Following incubation, cells were washed twice with PBS to remove excess dye and FluoroBrite DMEM (Gibco) was added prior to imaging with a Zeiss Cell Observer Spinning-Disk microscope with an excitation wavelength of 488 nm (oxidized C11-BODIPY) and 590 nm (reduced C11 -BODIPY). The acquired images were processed in ImageJ to measure mean fluorescence intensities for both channels. The level of lipid peroxidation was expressed as the ratio of oxidized to reduced C11-BODIPY fluorescence intensity.

### Seahorse analysis of mitochondrial function

Mitochondrial function was assessed by measuring the oxygen consumption rate (OCR) using a Seahorse XFe96 extracellular flux analyzer (Seahorse Biosciences) as previously described (35). Data were processed and analysed using Agilent Seahorse Wave Desktop software version 2.6.3.5. For this assay, patient fibroblasts were cultured in 96-well Seahorse plates at a density of 3 × 10^4^ cells per well and incubated overnight at 37 °C and 5% CO_2_. On the day of assay, sensor calibration was performed according to the manufacturer’s instructions. One hour before the assay, culture media was replaced with FBS, sodium bicarbonate, and phenol red-free RPMI medium, and cells were kept in a CO_2_-free incubator at 37 °C. Mitochondrial function was probed by the sequential addition of oligomycin (1 μM), FCCP (1 μM, and a mixture of rotenone (1 μM), and antimycin A (1 μM). Oligomycin (Oligo) inhibits ATP synthase (Complex V), carbonyl cyanide-4-(trifluoromethoxy)phenylhydrazone (FCCP) uncouples oxidative phosphorylation to reveal maximal respiratory capacity, and rotenone/antimycin A (Rot/AA) inhibit Complex I and III, respectively, to determine non-mitochondrial respiration. Maximal respiration was calculated as the difference between the maximum OCR after FCCP injection and the non-mitochondrial OCR measured after the addition of rotenone and antimycin A. Spare respiratory capacity was calculated as the difference between basal and maximal respiration. All experiments were normalized to total DNA content, quantified using Crystal violet staining.

### Total RNA isolation, cDNA generation, and quantitative real-time RT qPCR

Fibroblasts were cultured on 10 cm dishes under standard conditions. At ∼80% confluence, cells were harvested directly into 1 ml of TRIzol reagent (Cat. no. 15596026, Invitrogen), and total RNA was extracted following the manufacturer’s instructions. Nine hundred nanograms of purified RNA were reverse transcribed using the High-Capacity cDNA Reverse Transcription Kit (Cat. no. 4368814, Applied Biosystems). Quantitative real-time PCR was performed on a Bio-Rad CFX96 Touch Real-Time PCR Detection System (Bio-Rad) using TaqMan Fast Advanced Master Mix (Cat. no. 4444557, ThermoFisher Scientific), and TaqMan Gene Expression Assays (Cat. no. 4331182, ThermoFisher Scientific) for CHKA (RRID: Hs00957878_m1), CHKB (RRID: Hs01925200_s1), CNSK2A2 (RRID: Hs00751002_s1), CPT1B (RRID: Hs03046298_s1), PPARA (RRID: Hs00947536_m1), and PPARB/D (RRID: Hs04187066_g1). All reactions were performed in triplicate, and data were analyzed using LightCycler 96 Instrument Software, Version 1.1.1.

### SDS-PAGE and immunoblotting

Cells were lysed in RIPA buffer (50 mM Tris pH 8.0, 150 mM NaCl, 0.1% SDS, 1% sodium deoxycholate, 1% Triton X-100 and complete protease inhibitor cocktail). Protein concentration of the samples was determined using a Bicinchoninic acid assay (ThermoFisher, 23,227) using a bovine serum albumin standard as described by the manufacturer. Proteins were resolved by sodium dodecyl sulfate–polyacrylamide gel electrophoresis (SDS–PAGE) and transferred to nitrocellulose membranes (0.45 μm pore size, Amersham Protran). Membranes were blocked with Odyssey blocking solution for 1 h at room temperature and incubated with the appropriate primary antibodies diluted in Odyssey blocking solution at 4 °C overnight. Membranes were then washed in TBS-T three times for 5 minutes each and incubated with appropriate secondary antibodies in Odyssey blocking solution for 1 h at room temperature. Membranes were then washed in TBS-T three times for 5 minutes each, and protein bands were visualized using an Odyssey imaging system and bands densities quantified using FIJI software (NIH).

### Antibodies

The following primary antibodies and dilutions were used for immunoblotting: Anti-PPARA (Abcam, Cat# ab61182; used at 1:1000 dilution), Anti-PPARB (Thermo Fisher Scientific, Cat# PA1-823A; used at 1:1000 dilution), Anti-CPT1B – (Abcam, Cat# ab134988; used at 1:1000 dilution), Anti-GAPDH (1:1000, Cell Signaling Technology, Cat# 2118), Anti-CCTα (rabbit polyclonal, Cell Signaling Technology, Cat. #4454; used at 1:1000 dilution), and Anti-β-actin, Sigma, Cat# A5441; used at 1:5000 dilution). Secondary antibodies were Anti-rabbit IRDye-800, 1:20,000, LI-COR Biosciences, Cat# 926 to 32211 and Anti-mouse IRDye-680, 1:20,000, LI-COR Biosciences, Cat# 926 to 68070.

### Statistical analyses

Data are shown as mean ± SD from three independent experiments. Comparison between groups was done by one-way ANOVA followed by the Tukey’s or Dunnet’s test for multiple comparisons. *p* values < 0.05 were considered significant. All statistical analyses and graphs were generated using GraphPad Prism (Version 10.2.0).

## Data availability

All the data are contained within the manuscript or the supporting information.

## Supporting information

This article contains supporting information.

## Conflict of interest

The authors declare that they have no conflicts of interest with the contents of this article.
