# Supplementary material for: Neurodevelopmental disease-causing variants in choline kinase CHKA gene couple phosphatidylcholine synthesis to oxidative stress damage and disease etiology
Source: J Biol Chem. 2025 Nov 25;302(1):110983. doi: 10.1016/j.jbc.2025.110983 (PMC12800699; doi:10.1016/j.jbc.2025.110983)
Supplement: Supplementary Material 1 [file mmc1.pdf]

Supplemental data

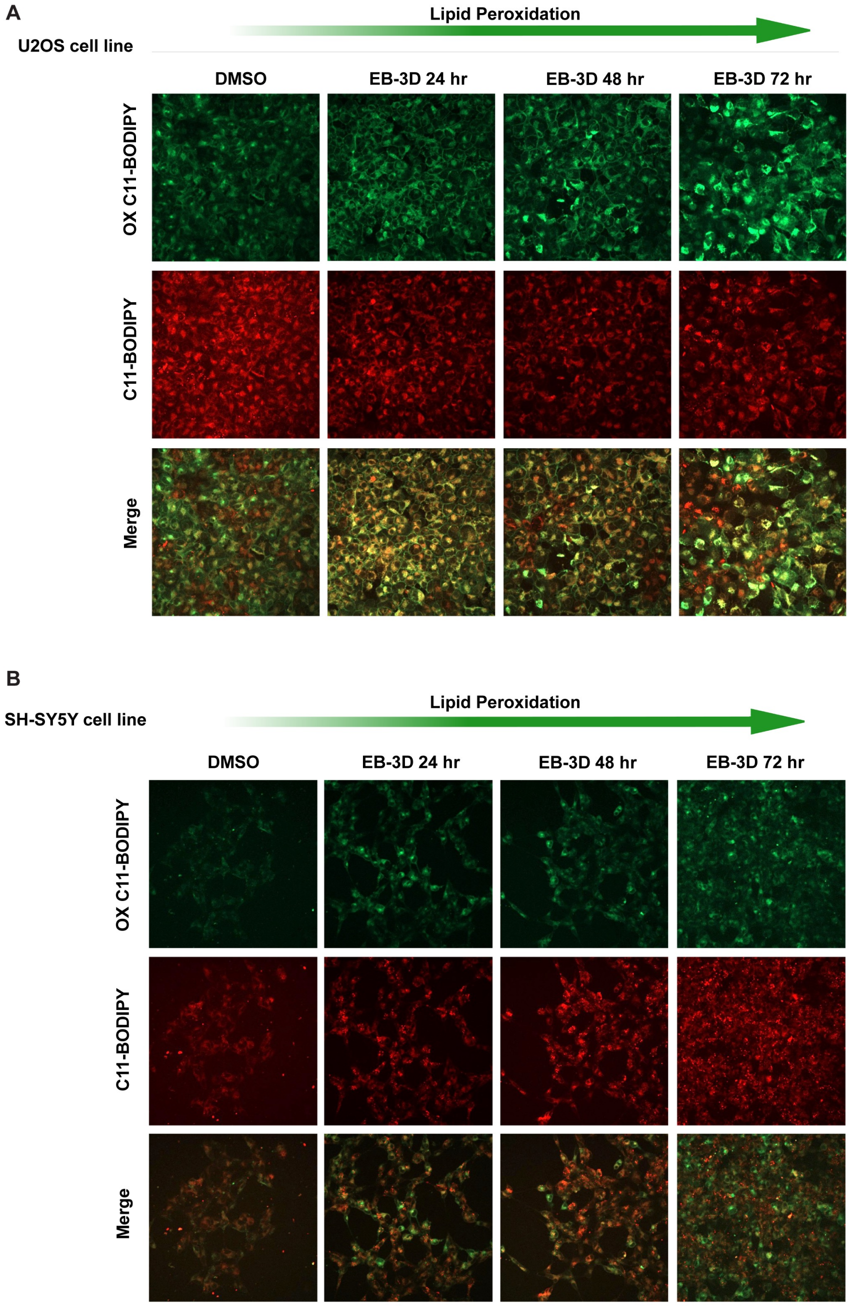

Supplementary Fig. 1. Treatment with the CHKA inhibitor EB-3D led to dose-dependent increase in lipid peroxidation. Representative images of U2OS cells **(A)** and SH-SY5Y **(B)** treated with EB-3D over 72-hours and challenged with ML162, a covalent glutathione peroxidase 4 (GPX4) inhibitor for 60 min and stained with BODIPY C11 as a measure of lipid peroxidation.
